# Severe Guillain-Barré syndrome after mRNA COVID-19 vaccination with rapid deterioration and recovery: a case report

**DOI:** 10.1093/omcr/omag153

**Published:** 2026-08-11

**Authors:** Raees Samli, Bara’a Al-Diri, Eyad Jamileh, Taghread Hudaib

**Affiliations:** Department of Medicine, Royal Blackburn Hospital, East Lancashire Hospitals NHS Trust, Haslingden Road, Blackburn, BB2 3HH England, United Kingdom; Department of Medicine, Royal Blackburn Hospital, East Lancashire Hospitals NHS Trust, Haslingden Road, Blackburn, BB2 3HH England, United Kingdom; Department of Medicine, Royal Blackburn Hospital, East Lancashire Hospitals NHS Trust, Haslingden Road, Blackburn, BB2 3HH England, United Kingdom; School of Medicine, Dentistry and Biomedical Sciences, Queens University Belfast, University Road, Belfast BT9 7BL, Northern Ireland, United Kingdom; School of Pharmacy, University of Lincoln, Brayford Pool, Lincoln, LN6 7TS, England, United Kingdom

**Keywords:** Guillain–Barré syndrome, COVID-19 vaccination, mechanical ventilation, critical care, case report

## Abstract

Guillain-Barré syndrome (GBS) is an acute immune-mediated polyneuropathy characterised by rapidly progressive weakness, sensory disturbance, and autonomic dysfunction, often requiring intensive supportive care. While most cases follow gastrointestinal or respiratory infections, rare cases have been reported after vaccination. A 72-year-old man developed progressive symmetrical weakness and areflexia three weeks after receiving an mRNA COVID-19 vaccine. His condition deteriorated to bulbar dysfunction and respiratory failure, requiring intubation, tracheostomy, and prolonged ventilatory support. Cerebrospinal fluid analysis demonstrated albuminocytologic dissociation, while neuroimaging excluded structural pathology. Intravenous immunoglobulin was initiated promptly, followed by intensive multidisciplinary rehabilitation, including hydrotherapy. He gradually improved, was successfully decannulated, and transferred to a rehabilitation facility. This case highlights a rare possible post-vaccination association while emphasising that causality remains uncertain. Early recognition, close respiratory monitoring, and comprehensive rehabilitation are essential, as meaningful recovery is achievable even after severe disease.

## Introduction

Guillain-Barré syndrome (GBS) is an acute immune-mediated polyneuropathy characterised by rapidly progressive weakness and areflexia, with variable sensory or autonomic involvement [1]. It is the most common cause of acute flaccid paralysis worldwide, with an incidence of 1–2 cases per 100 000 annually [1]. Its pathogenesis is typically post-infectious, involving molecular mimicry whereby antibodies cross-react with peripheral nerve gangliosides, causing demyelination or axonal injury [2]. Rare cases have also been reported following vaccination [3, 4]. During global COVID-19 vaccination programmes, GBS has emerged as a rare potential neurological complication [5–9].

We present a severe case of GBS developing three weeks after mRNA COVID-19 vaccination, notable for rapid progression to respiratory failure requiring prolonged ventilatory support, followed by favourable recovery. This case highlights the importance of early recognition, immunotherapy, and multidisciplinary rehabilitation. The CARE Checklist is attached as Supplementary Material S1.

## Case presentation

A previously well and independent 72-year-old man with no past medical history presented to the emergency department with new-onset left lower limb weakness, first noticed while performing routine daily activities. He had received an mRNA COVID-19 vaccine three weeks prior and reported a one-week history of lethargy and non-specific flu-like symptoms preceding presentation. Neurological examination demonstrated reduced power and sensation in all four limbs, with asymmetrical weakness more pronounced on the left side and greater involvement of the lower limbs. The patient also reported intermittent paraesthesia in the upper limbs. Deep tendon reflexes were absent in the lower limbs, while plantar responses were normal. There were no gastrointestinal symptoms or other focal neurological deficits reported. At presentation, there was no evidence of respiratory compromise.

Initial blood investigations were largely unremarkable. Blood and sputum cultures remained negative after incubation, with no bacterial organisms isolated, indicating no evidence of bacteraemia from culture-based testing. Viral serology (hepatitis B, hepatitis C, HIV, CMV, EBV) and respiratory viral PCR testing for influenza and SARS-CoV-2 were all negative. Computed tomography (CT) of the brain and magnetic resonance imaging (MRI) of the spine excluded an intracranial or spinal cause for the presentation. Faecal cultures and culture-independent diagnostic tests, including tests for *Campylobacter Jejuni*, were not performed as there was no clinical indication or history to suggest gastrointestinal involvement. Cerebrospinal fluid (CSF) analysis demonstrated a white cell count of 4 cells/μL and an elevated protein concentration of 1.12 g/L, consistent with albuminocytological dissociation [7]. CSF bacterial culture, and viral PCR testing for herpes simplex virus type 1 (HSV-1), herpes simplex virus type 2 (HSV-2), varicella-zoster virus (VZV), enterovirus and parechovirus, were negative. Electrophysiological studies were not performed as this would have required transfer to a regional centre. In the context of progressive symmetrical weakness with areflexia and supportive CSF findings, a clinical diagnosis of GBS was established, fulfilling Brighton Level 2 diagnostic certainty. Given the high clinical suspicion for GBS, intravenous immunoglobulin (IVIg) was initiated at a total dose of 2 g/kg administered over five days, consistent with established immunotherapeutic management of GBS, and the patient was admitted to the intensive care unit (ICU) for close monitoring [8]. Despite treatment, neurological function deteriorated rapidly. By day 3, muscle strength had declined to 1/5 in all four limbs. On day 4, the patient developed confusion and dysphagia, necessitating nasogastric tube insertion for enteral feeding and designation as nil by mouth due to an unsafe swallow. By day 5, the patient exhibited increasing oxygen requirements, indicating evolving respiratory compromise. In view of progressive neuromuscular weakness and impending respiratory failure, the patient was sedated, intubated, and mechanically ventilated. In anticipation of a prolonged ventilatory course, an early percutaneous tracheostomy was performed. Sedation was discontinued by day 7, and the full course of intravenous immunoglobulin was completed. This was followed by a prolonged, structured respiratory weaning process alongside gradual neurological recovery, as outlined in Table 1. Care was coordinated through a designated lead critical care consultant assigned to this patient, who worked closely with the multidisciplinary team, including nursing staff, physiotherapists, occupational therapists, speech and language therapists, dieticians, psychologists, and pharmacists, to optimise daily management and rehabilitation.

**Table 1:** Key milestones in the patient’s respiratory and functional recovery during intensive care.

Day post-presentation	Progression
10–17	Unable to tolerate cuff-down; intermittent plugging and desaturation episodes.
18	Focus on chest physiotherapy, aiming for 4 × 15-minute cuff-down sessions daily.
20	No meaningful motor movement. Hallucinations and significant fatigue noted.
24	Weaning progress stagnant due to physiotherapy fatigue and secretion load.
30	Rapid cuff-down progress; longer cuff-down periods with breaks as required.
36	Aiming for 24-hour cuff-down.
40	Continuous positive airway pressure (CPAP) sprints commenced.
43	Regaining some strength with independent sitting balance.
45	Achieved 8 hours of zero-pressure support.
55	Achieved 16 hours of zero-pressure support. No functional movements such as raising hands to face.
60	Spending longer periods of time without pressure support on passive humidification bib.
63	First hydrotherapy session; managed 3–4 shuffling steps in the pool.
67	On passive humidification bib for 24 hours.
69	Tracheostomy decannulated.
70	Further steps in hydrotherapy pool, 12 meters covered, along with enhanced range of upper body movements. Improved buoyancy.
77	Hydrotherapy session: performing squats, upper body rotations, and kicking legs.
80	Discharged from critical care and transferred to rehabilitation facility.

## Discussion

This case describes a severe and rapidly progressive presentation of GBS temporally associated with recent COVID-19 vaccination, requiring early mechanical ventilation and prolonged critical care support, followed by favourable functional recovery.

GBS is an acute immune-mediated polyradiculoneuropathy characterised by progressive weakness, areflexia, and variable sensory symptoms. In this case, diagnosis was established clinically and supported by cerebrospinal fluid albuminocytological dissociation, fulfilling Brighton Level 2 diagnostic certainty [6]. Although electrophysiological studies were not performed due to logistical challenges, the rapid progression to quadriparesis and early respiratory compromise raise the possibility of an axonal variant, often associated with more severe disease [5–10].

The absence of a preceding infective trigger prompted consideration of vaccination as a potential precipitating factor. While epidemiological studies suggest a small increased risk of GBS with some vaccine platforms, particularly adenoviral vector vaccines, evidence for mRNA vaccines remains inconsistent [9]. Proposed mechanisms include immune-mediated cross-reactivity following spike protein expression, although causality remains unproven [10–13]. Symptom onset three weeks post-vaccination is biologically plausible within adaptive immune activation timelines [9–13].

This case is notable for several features. First, the patient demonstrated hyperacute deterioration to profound quadriparesis within three days, followed by bulbar dysfunction and respiratory failure by day 5 despite prompt intravenous immunoglobulin, suggesting a severe subtype. Second, early anticipatory critical care management, including timely intubation and tracheostomy, facilitated structured respiratory weaning and may have contributed to the favourable outcome. Third, despite profound neurological impairment, meaningful recovery occurred through multidisciplinary rehabilitation, including hydrotherapy, which may support mobilisation and muscle reconditioning [10, 11].

Previous reports of post-vaccination GBS demonstrate substantial variability, ranging from mild disease to fulminant respiratory failure. This case highlights the importance of host-specific factors in disease trajectory. Early recognition, immunotherapy, vigilant respiratory monitoring, and coordinated rehabilitation remain critical [12, 13].

## Conclusion

This case describes a rare and severe presentation of GBS occurring three weeks following mRNA COVID-19 vaccination, characterised by rapid progression to respiratory failure and prolonged recovery. Although causality cannot be definitively established, the temporal relationship and absence of alternative triggers suggest a possible vaccine-related immune-mediated mechanism. Continued surveillance and mechanistic research are essential, although such events remain exceedingly rare and the benefits of COVID-19 vaccination continue to far outweigh associated risks.

## Supplementary Material

CARE_GBS_checklist_omag153
